# Classification of 16S rRNA reads is improved using a niche-specific database constructed by near-full length sequencing

**DOI:** 10.1371/journal.pone.0235498

**Published:** 2020-07-13

**Authors:** Phillip R. Myer, Tara G. McDaneld, Larry A. Kuehn, Keith D. Dedonder, Michael D. Apley, Sarah F. Capik, Brian V. Lubbers, Gregory P. Harhay, Dayna M. Harhay, John W. Keele, Madison T. Henniger, Brooke A. Clemmons, Timothy P. L. Smith

**Affiliations:** 1 Department of Animal Science, University of Tennessee Institute of Agriculture, University of Tennessee, Knoxville, TN, United States of America; 2 USDA-ARS, U.S. Meat Animal Research Center, Clay Center, NE, United States of America; 3 College of Veterinary Medicine, Kansas State University, Manhattan, KS, United States of America; Tierarztliche Hochschule Hannover, GERMANY

## Abstract

Surveys of microbial populations in environmental niches of interest often utilize sequence variation in the gene encoding the ribosomal small subunit (the 16S rRNA gene). Generally, these surveys target the 16S genes using semi-degenerate primers to amplify portions of a subset of bacterial species, sequence the amplicons in bulk, and assign to putative taxonomic categories by comparison to databases purporting to connect specific sequences in the main variable regions of the gene to specific organisms. Due to sequence length constraints of the most popular bulk sequencing platforms, the primers selected amplify one to three of the nine variable regions, and taxonomic assignment is based on relatively short stretches of sequence (150–500 bases). We demonstrate that taxonomic assignment is improved through reduced unassigned reads by including a survey of near-full-length sequences specific to the target environment, using a niche of interest represented by the upper respiratory tract (URT) of cattle. We created a custom Bovine URT database from these longer sequences for assignment of shorter, less expensive reads in comparisons of the upper respiratory tract among individual animals. This process improves the ability to detect changes in the microbial populations of a given environment, and the accuracy of defining the content of that environment at increasingly higher taxonomic resolution.

## Introduction

A critical step in 16S-based microbiome research is the assignment of microbial taxonomy. Currently, most research utilizes public 16S reference databases, such as Greengenes, SILVA, RDP, or GenBank, to assign taxonomy to sequence data [1–4]. However, these databases may pose limitations to taxonomic assignment due to the large amount and length of sequences available as well as the variable quality of data. For example, 43% of full-length 16S small-subunit rRNA gene sequences available in the GenBank database were identified as chimeras and unable to be classified, resulting in low-quality data that can ultimately alter taxonomic assignment [1]. Unlike GenBank, Greengenes and SILVA utilize a chimera-checking and alignment algorithm for quality filtering [5, 6]. Even with these quality-checking programs, these public 16S reference databases contain between 0.2% to 2.5% mislabeled sequences, with errors associated with knowledge bases that have misidentified species or misidentified sequence data that result in incorrectly assigned taxa [7]. When these reference databases were compared among each other using taxonomy annotations provided by RDP to assess taxonomic assignment, Greengenes and SILVA significantly differed from each other, with an error rate around 17% [8]. Not only do these public databases contain significant error rates but also thousands of sequences. In August 2007, SILVA had 546,521 sequences available [6]; now, SILVA reports having over 6 million aligned rRNA sequences [9]. The sequences available in these databases are not always full-length sequences as well, and have historically focused on amplicons of hypervariable regions [10]. The classification accuracy differs among hypervariable regions, and can negatively impact taxonomic assignment based on the regions referenced [11]. Greengenes provides full-length and chimera-checked 16S rRNA gene sequences, created from multiple curators who provide their sequences from a single study. However, among many curators, there can be multiple incongruencies in taxonomic nomenclatures at the phylum level (1). Ultimately, this has the potential to lead to errors in assignment when using these public databases. In addition, the extensive number of sequences to process can have a large impact on computational resources [12]. Therefore, to assist with the limitations associated with public 16S reference databases, using custom 16S reference databases developed for specified microbial niches has the potential to improve taxonomic assignment while reducing the computational load that is associated with public 16S reference databases. With increased performance, researchers can more accurately investigate the impact of microbial communities and understand their roles in specific environments, particularly with diseases that are high-impact in certain industries.

Bovine respiratory disease (BRD), or shipping fever, substantially impacts the U.S. beef cattle industry, with economic losses exceeding $1 billion annually [13, 14]. Calf mortality, veterinary and/or treatment costs, and labor are among the primary contributors to the financial burden of BRD on the industry. The manifestation of the disease can result in reduced growth and reproductive performance [15, 16], subsequently affecting long-term herd performance and longevity. A variety of complex factors can be involved with BRD pathogenesis that involve host elements, environmental elements, inadequate management and housing, as well as viral and bacterial pathogens, such as *Mannheimia haemolytica* and *Mycoplasma bovis* [17, 18]. Yet, the presence of these pathogens in the upper respiratory tract (URT) of cattle may not necessarily result in disease [19]. Indeed, microbes such as *M*. *haemolytica* are opportunistic and result in diseased states when the animal is exposed to environmental stressors such as transport, weaning, or viral pathogens [20]. As BRD is reliant on opportunistic conditions, research is beginning to focus on the association of these bacterial pathogens with the animal’s nasopharyngeal microbiome [21–23]. Understanding these associations are key in determining the role of the microbiome on BRD, and how microbial community dysbiosis may impact the incidence of respiratory disease in cattle.

Currently, microbiome research efforts with regard to BRD have focused on characterizing the microbiome of the bovine nasal cavity in normal and diseased states in varying stages of production [23–25]. These community-based studies conducted 16S rRNA analyses utilizing short-read technologies. However, as is similar in other fields of animal agriculture microbiome research, typically a large quantity of sequence reads is left unassigned (approximately 10–20% in BRD research) [26] and/or operational taxonomic units (OTUs) with low sequence counts are removed from analyses. These shortcomings decrease the power of analyses to detect small, potentially important variations in microbial community structure and function that may have phenotypic or disease-related impacts. Greater use of community-based microbial databases has been a more practical option in defining accurate microbiomes [27, 28]. Utilizing niche-specific microbial databases developed from full-length or near-full-length 16S reads for short-read 16S analyses would aid in microbial identification and further limit unassigned taxa [29, 30]. In BRD research this is critical, as optimizing the ability to accurately and comprehensively investigate the nasopharyngeal microbiome permits researchers with greater opportunities to define the complex interactions in this multifactorial disorder. In this report, we describe the development of a 16S rRNA sequence database for the bacterial community present in the URT of cattle, to provide improved assignment of taxa within the bovine URT and as a site-specific tool for examining microbiome dysbiosis involved in BRD.

## Materials and methods

### Database construction

#### Animal selection and sampling

This study was approved and carried out in accordance with the recommendations of the Kansas State University and U.S. Meat Animal Research Center Institutional Animal Care and Use Committees. Animal selection and sampling procedures for the “KSU” population were conducted as described in DeDonder and colleagues [31]. The KSU population consisted of a total of 180 cattle with an initial body weight ranging from 164–269 kg, obtained from Athens, Tennessee (*n* = 60), Maryville, Missouri (*n* = 60), and Richmond, Kentucky (*n* = 60). Comingled steers and bulls were acquired from multiple origins and mixed breeds to obtain a broad spectrum of variability for bacterial classification. The cattle were transported to a feeding facility in Kansas and housed in open-air, dirt floor, group housing pens. Cattle were randomly assigned one of two treatments upon arrival at the facility, receiving either mock treatment saline at 2 mL / 49.9 kg or metaphylactic gamithromycin at the dose of 2 mL / 49.9 kg subcutaneously in the neck. Additional information regarding vaccines, growth implants, and diets are further described according to DeDonder and colleagues [31]. Animal selection and sampling procedures for the “USMARC” population were conducted as described by McDaneld et al. (24). Samples were collected in the years 2010–2012 from animals belonging to advanced generations of the U.S. Meat Animal Research Center GPE (Germplasm Evaluation Program; [32]) herd, Clay Center, Nebraska. Approximately 800 animals were produced in multiple-sire matings of crossbred cows to F1 and purebred bulls from various breeds, consisting of variable fractions of 18 breeds: Angus, Hereford, Red Angus, Brahman, Charolais, Gelbvieh, Limousin, Simmental, Brangus, Beefmaster, Shorthorn, Maine Anjou, Santa Gertrudis, Chiangus, Salers, Braunvieh, South Devon, and Tarentaise. As a number of factors affect the microbial communities of the nares in cattle with regard to BRD, such as environment, management, sex, breed, and clinical treatment [23, 33], this optimized strategy and diverse mix of cattle was used to establish the bacterial Bovine URT 16S rDNA database for cattle with BRD.

The KSU population was sampled multiple times, including at the sale barn prior to departure on transport trucks, during unloading upon arrival at the feedlot, and at least twice more during the 28-day feeding period. Some animals had additional sample collected if they were diagnosed as potential BRD cases. Sampling was conducted by deep nasopharyngeal swab inserted through the nares by a trained veterinarian as described [17]. Briefly, the double guarded, sterile uterine swab was inserted into the nasal cavity to the point of resistance against the swab, indicating nasopharyngeal tonsilar tissue. The swab was rotated to obtain sample from the pharyngeal tissues. During swab removal, the guarded sleeve protected the swab from contamination. One swab was inserted in each nostril, and kept separate during transport. The swabs were stored in liquid Amies media and sent on ice to the USDA-US Meat Animal Research Center (Clay Center, NE) for same-day processing (approximately 4 hours in transit). The tubes containing media and swabs were vortexed for 30 seconds to displace bacteria still stuck on the swabs, and then the swabs were removed. Aliquots from the tubes of independent swabs from both nares of each animal were taken, and then the remainder of each pair of tubes was combined into a single animal-by-time-point combined sample. Glycerol was added to 20% final solution to each combined sample and independent aliquot, mixed by inversion, allowed to stand for 5 minutes on ice, and then stored at -80°C.

The USMARC population was sampled using 6-inch nasal swabs of calves at initial vaccination (approximately 40 days of age), preconditioning (approximately 130 days of age), weaning (approximately 150 days of age), and if the calf was diagnosed with BRD during a 1-5-week period after weaning. For sampling, the nose of the animal was wiped cleaned with a single-use towel if fecal material was present. The unguarded 6-inch nasal swab was then gently inserted into the nasal cavity at an approximate depth of 6 inches. The nasal swab was then rotated and removed. After collection of the sample, 6-inch nasal swabs were placed in buffered peptone water with 12% glycerol, drop frozen in liquid nitrogen directly after collection, and stored at -80°C.

#### DNA extraction, amplification, and sequencing of database samples

For the KSU samples, a 1mL aliquot of sample was placed into LoBind tubes (Eppendorf, Hamburg, Germany) containing Nuclei Lysis Solution from the Promega Wizard Genomic DNA Purification Kit (Madison, WI, USA) and an isopropanol precipitation was performed on ice using a total of 600μL of 100% isopropanol. Samples were then centrifuged at 14,500 x *g* for 2 min. at 4°C. A 600μL volume of cold Nuclei Lysis buffer was added to the pellet, vortexed, and incubated at 80°C for 7 min. Samples were then transferred to QiaShredder (Qiagen, Hilden, Germany) tubes and centrifuged at 14,500 x *g* for 5 min. at 4°C. Samples were then vortexed and incubated at 80°C for 5 min. Following incubation at room temperature for 10 min, 3μL of RNAse solution (Promega Wizard Genomic DNA Purification Kit) was added to the sample, mixed via inversion, and incubated at 37°C in a water bath for 30 min. Following incubation at room temperature for 10 min, 200μL of Protein Precipitation Solution (Promega Wizard Genomic DNA Purification Kit) was added to the sample, vortexed, and placed on ice for 5min. Samples were centrifuged at 14,500 x *g* for 3 min. at 4°C, supernatant transferred to a new tube, and 600μL of 100% isopropanol added to the sample and mixed via inversion. Another round of centrifugation at 14,500 x *g* for 2 min. at 4°C was completed, supernatant removed, and 600μL of 70% ethanol was added to the sample. A final centrifugation occurred at 14,500 x *g* for 2 min. and the 70% ethanol was removed via aspiration. The nucleic acid pellet was dried at room temperature for 15 min. and re-suspended in 50μL of DNA rehydration solution (Promega Wizard Genomic DNA Purification Kit). DNA quality was checked via electrophoresis in a 2% (w/v) agarose gel in TBE buffer (1X), and concentration was determined using a Nanodrop 1000 spectrophotometer (ThermoScientific, Wilmington, USA).

For the USMARC samples, total DNA was extracted from each swab using a commercial kit (PowerSoil DNA kit; Qiagen, Germantown, MD) as directed by the manufacturer, and initial DNA quantity was evaluated with a DNA spectrophotometer (DeNovix DS-11 FX Series; Wilmington, DE). Equal amounts of DNA from each swab were then pooled within collection year and time point of sampling (initial vaccination, preconditioning, weaning, after weaning) and were chosen for amplification of the 16S ribosomal RNA (rRNA) gene. There were 114 pools sequenced with long reads representing over 800 animals.

DNA library preparation was performed by polymerase chain reaction (PCR) amplification similar to Myer and colleagues [29]. PCR amplification and DNA library preparation of the V1–V8 hypervariable regions of the bacterial 16S rRNA gene were performed using universal primers 27F (5'- AGAGTTTGATCCTGGCTCAG) and 1392R (5'-GACGGGCGGTGTGTAC). Amplification consisted of 20 cycles with an annealing temperature of 58°C and extension time of 90 seconds. Products were purified using AmPure^®^ bead purification (Agencourt, Beverly MA), converted to libraries using Template Preparation Kit v1.0 (Pacific Biosciences), and quantified by the PicoGreen^®^ dsDNA quantitation kit (Invitrogen, Carlsbad, CA) and by real-time PCR on the LightCycler 480 system (Roche, Mannheim, Germany). Libraries were sequenced on the Pacific Biosciences RSII instrument (Pacific Biosciences, Menlo Park, CA).

#### Bovine URT database sequence data analyses, development, and phylogenetic analyses

All sequencing data were curated using mothur (v1.39.5) [34]. The consensus fastq files were parsed so that scores of zero were interpreted as corresponding to an ambiguous base call. Also, because the consensus sequence can be generated in the forward and reverse complement orientations, proper orientation was verified. Sequences were then filtered for quality (≥ Q30) using a rolling window approach (window = 30), homopolymers > 8 were discarded, any sequences with ambiguous bases were removed, and sequences that contained read lengths shorter than 1350bp or larger than 1400 were removed. Alignment was conducted against the SEED SILVA database (v128) [2]. Pre-clustering of aligned sequences was conducted at diffs = 14. De novo detection of chimeras was conducted using UCHIME [35]. Classification of denoised reads was performed against greengenes (v13_8_99) [1] using a naïve Bayesian classifier at a cutoff of 80. All reads were clustered with VSEARCH at a 97% similarity threshold. Following clustering, a consensus classification of OTUs using a 50% consensus clustering threshold was performed. Then, a taxonomic database consisting of the most abundant sequence of each OTU was constructed. Rarefaction curves examining sequencing depth were constructed in the Quantitative Insights Into Microbial Ecology (QIIME) bioinformatics pipeline, version 1.9.1 [36]. Using the whole database alignment, Newick-formatted, approximately maximum-likelihood phylogenetic trees were calculated and built in QIIME using FastTree [37]. Trees were viewed using Interactive Tree of Life (iTOL) [38]. These sequences are available from the NCBI Sequence Read Archive (SRA Accession PRJNA548468).

### Validation analyses

#### DNA amplification and sequencing

The 16S ribosomal RNA (rRNA) gene variable regions were amplified from additional pooled samples of the USMARC population. Three pooled samples were randomly chosen for sequencing and analyses. DNA was extracted as described above. Library preparation was performed using standard PCR (AccuPrime, Invitrogen, Carlsbad, CA) and primers that amplify variable regions 1 through 3 of the 16S rRNA gene [39]. Quality and quantity of the resulting 16S rRNA gene amplification was checked on the Fragment Analyzer (Advanced Analytical, Ankeny, IA) and then sequenced utilizing the MiSeq Illumina Sequencer (Illumina, San Diego, CA) with a MiSeq Reagent Kit v3 to generate 2x300 paired end reads. These sequences are also available from the NCBI Sequence Read Archive (SRA Accession PRJNA548468).

#### Sequence data analyses

All MiSeq sequencing data for validation analyses were curated using mothur (v1.39.5) [34]. Sequences were then filtered for quality (≥ Q30) using a rolling window approach (window = 30), homopolymers > 8 were discarded, any sequences with ambiguous bases were removed, and sequences that contained read lengths shorter than 300bp were removed. Alignment was conducted against the SEED SILVA database (v128). Pre-clustering of aligned sequences was conducted at diffs = 14. *De novo* detection of chimeras was conducted using UCHIME. Classification of denoised reads was performed against Greengenes (v13_8_99) [1] and SILVA (v128) (2) using a naïve Bayesian classifier at a cutoff of 80. For comparison, classification of denoised reads was also performed against the Bovine URT database. All reads were clustered with VSEARCH at a 97% similarity threshold. Following clustering, a consensus classification of OTUs using a 50% consensus clustering threshold was performed. Sequences and their resultant taxonomic classification that were successfully assigned by the Bovine URT database but unassigned against the Greengenes database were re-analyzed using the Bovine URT database. To accomplish this, unassigned Greengenes sequences were filtered from the FASTA and OTU tables to retain only those sequences that were unassigned. Utilizing the filtered FASTA and OTU tables, the classification of the filtered unassigned sequences was then performed against the Bovine URT database as described previously to ascertain differences in taxonomic assignment using the Bovine URT database. Phylum-level taxa were assessed for normality using the PROC UNIVARIATE procedure in SAS 9.4 (SAS Institute, Cary, NC). Normality was determined based on visual distribution of histograms and a Shapiro-Wilk statistic of ≥ 0.90. Those variables following a normal distribution were analyzed using one-way analysis of variance (ANOVA) for multiple independent groups [40] to compare among Bovine URT, SILVA, and Greengenes datasets. Those variables following a non-normal distribution were first ranked and then analyzed using a one-way ANOVA. Significance was determined at *P* ≤ 0.05 and tendencies were considered from 0.05 < *P* ≤ 0.10 and the Benjamini–Hochberg method used for multiple-testing corrections [41]. Taxonomic composition correlation between the database pipelines was conducted using Pearson correlation, where *P*-values were calculated using a two-sided test of significance using a t-distribution.

## Results

### Summary of database composition

**Fig 1:**
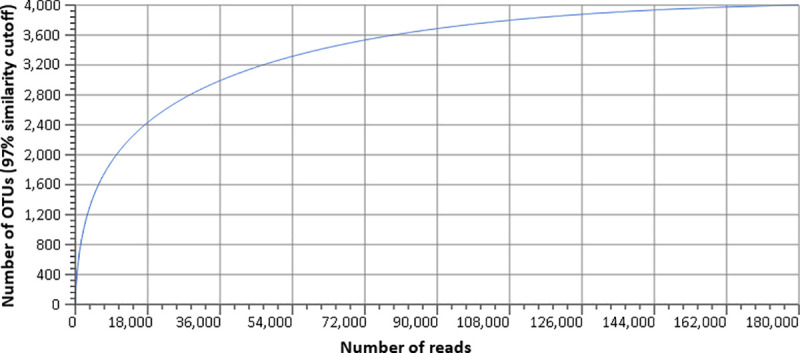
Rarefaction curve of annotated species richness within the Bovine URT bacterial 16S rDNA reference database.

A summary of database statistics is included in Table 1. A total of 4,364,029 reads were used for Bovine URT database development following pipeline QC, with 170,730 unique sequences totaling 235,106,702 bp with an average length of 1,377 ± 36 bp. A large quantity of the database consists of singletons, totaling 150,723 reads. This is due to the inclusion of unique reads, including ones with only 1bp difference. As a means to evaluate the completeness of detection of bacterial species, a rarefaction curve of annotated species richness was constructed and resulted in adequate read depth as depicted by the asymptote (Fig 1). Clustering the reads using presently known species resulted in a total number of 2,111 observed species (S1 File). Overall, the Bovine URT database contains a small number of abundant species, but a large quantity of rare species.

**Table 1:** Bovine URT database statistics.

Metric	Value
Number of Sequences	170,730
Average Sequence Length (bp^1^)	1,377 ± 36
Mean GC Content (%)	49 ± 4
Number of Species-Level OTU^2^	2,111
Number of Genus-Level OTU^2^	510

^1^BP: Base pairs

^2^OTU: Operational taxonomic units

**Fig 2:**
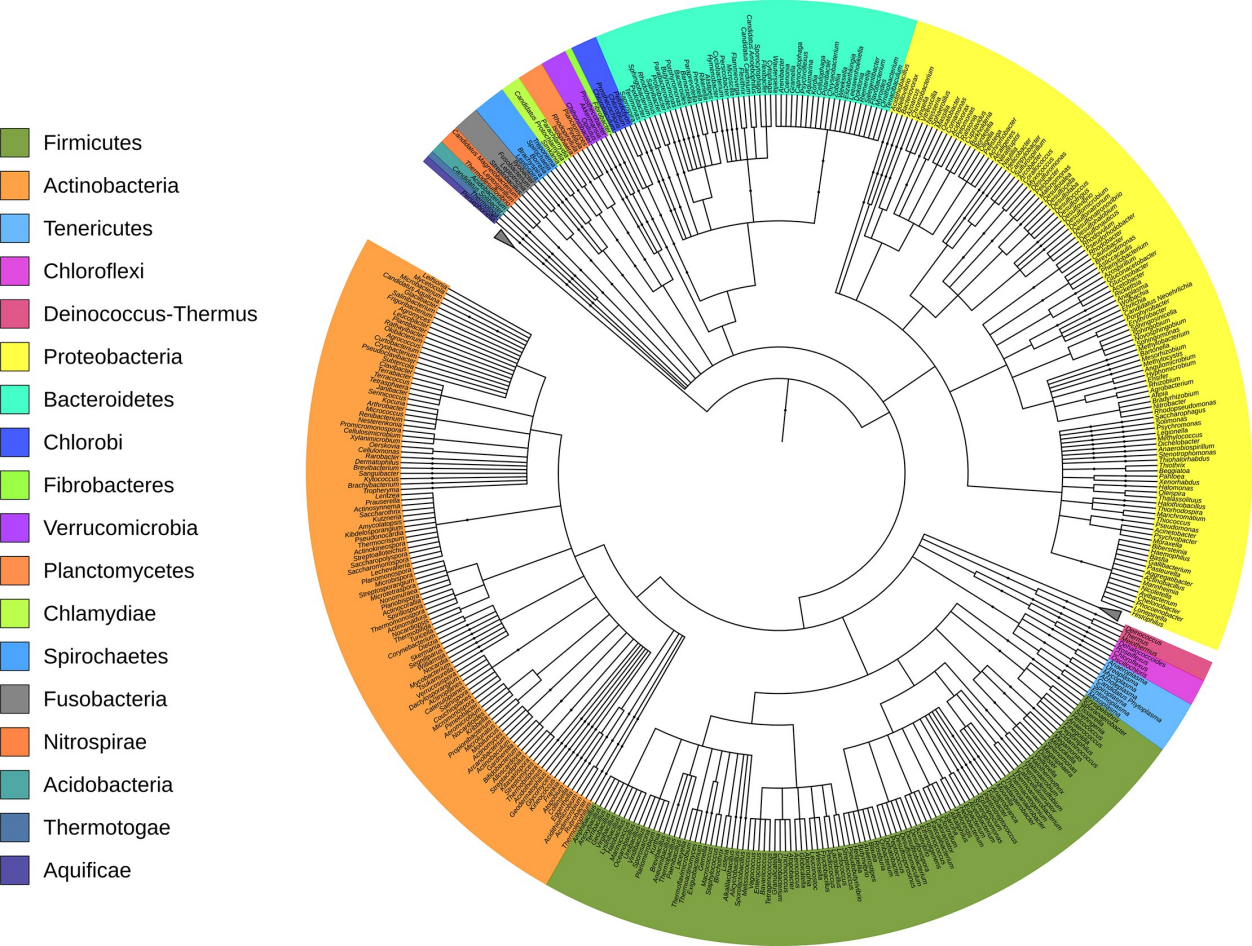
Circular maximum likelihood phylogenetic tree at the level of genus of the Bovine URT 16S rDNA reference database. Tree image was generated using ITOL. Genera are color-coded by phylum.

**Fig 3:**
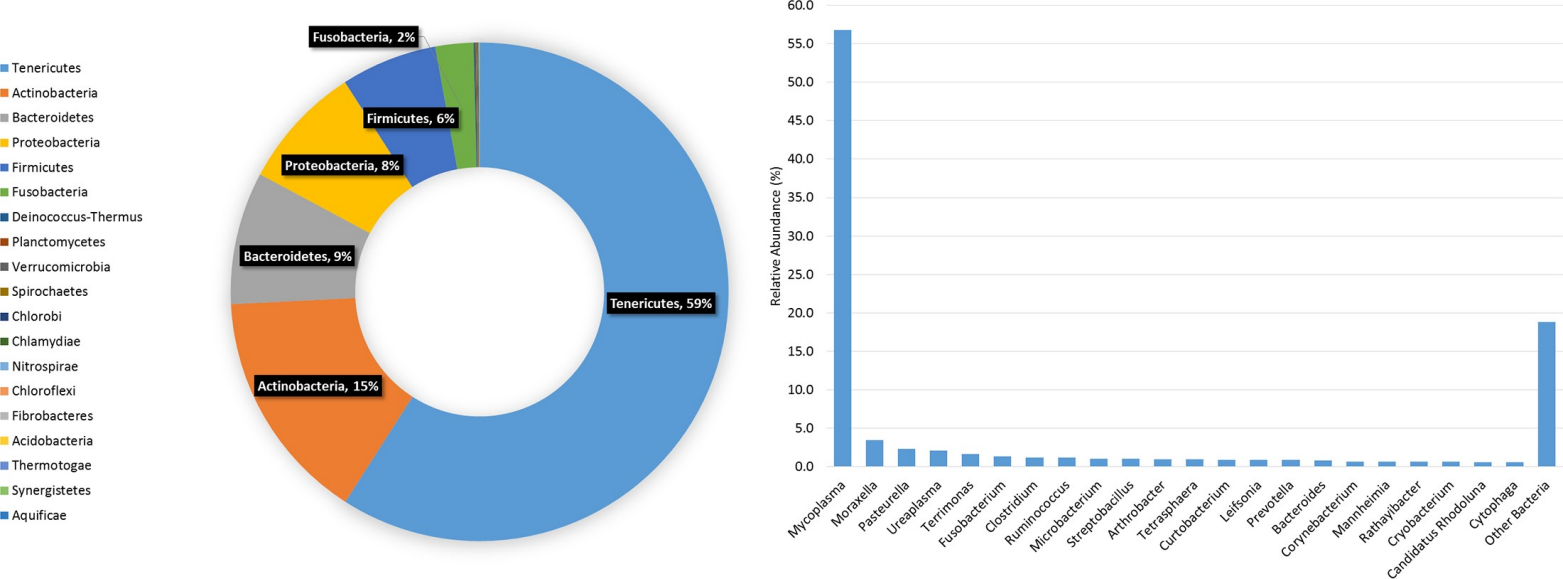
**Bacterial taxonomic profile of total 16S rDNA gene sequences in the Bovine URT reference database at the phylum level (donut chart; left) and genus level (bar chart; right).** Genus abundances classified by representation at ≥0.6% of total sequences.

A detailed phylogenetic tree of the Bovine URT bacterial 16S database at the level of genus is displayed in Fig 2. A detailed taxonomic distribution of the reads at the level of phylum and genus within the database are listed in Fig 3. *Mycoplasma* was the dominant genus, accounting for over 57% of the reads. The only other genus observed at ≥1% included *Moraxella* (3.4%), *Pasteurella* (2.3%), *Ureaplasma* (2.1%), *Terrimonas* (1.6%), *Fusobacterium* (1.4%), *Clostridium* (1.2%), *Ruminococcus* (1.2%), *Microbacterium* (1.1%), and *Streptobacillus* (1.0%). *Mannheimia* was present at 0.7% (S2 File). Only 793 sequences were unable to be identified (0.4%).

### Reference database performance

**Fig 4:**
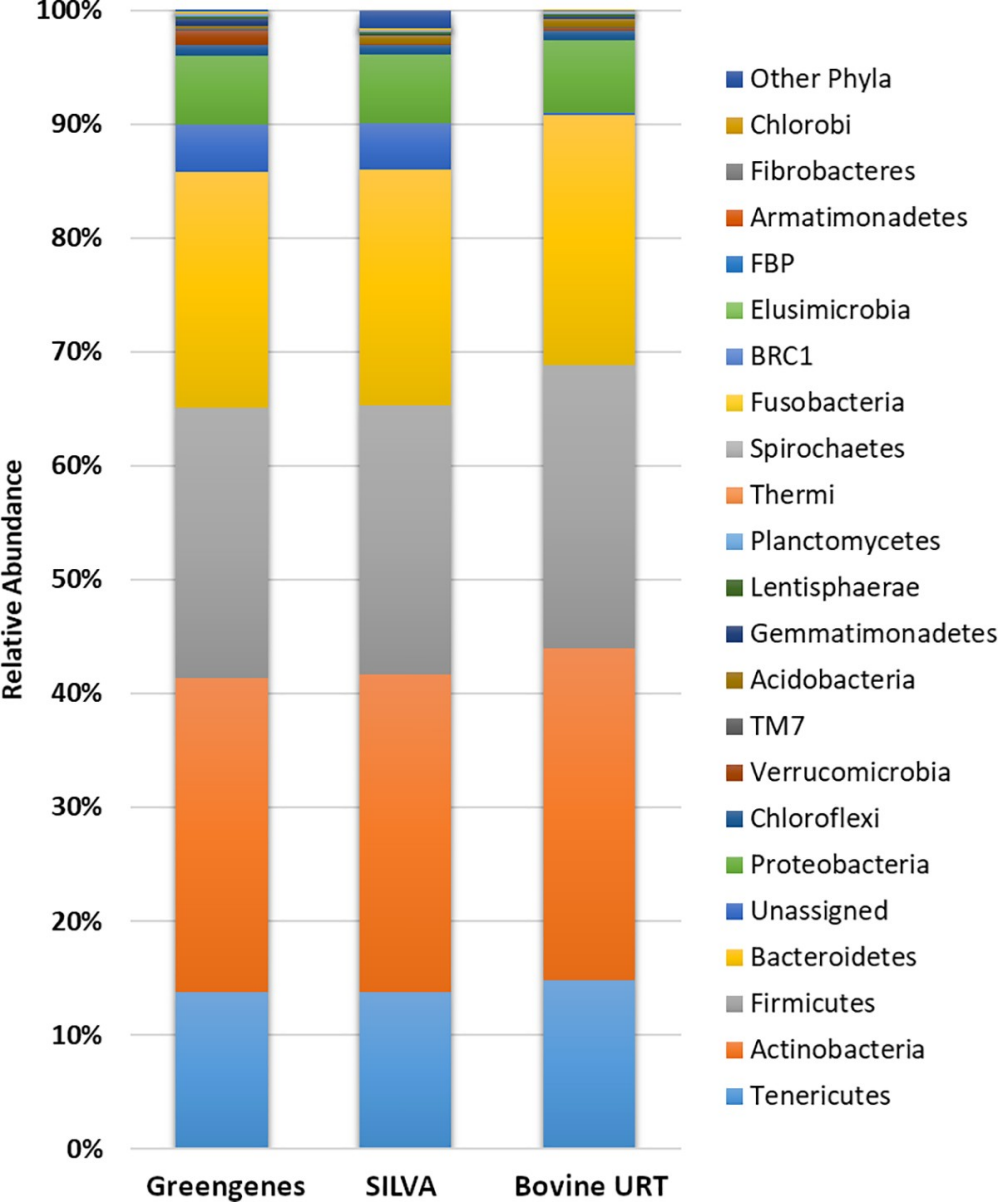
Relative abundance of bacterial phyla in the multiple diverse samples identified using the Greengenes, SILVA, and Bovine URT databases on small read 16S amplicon sequencing from the bovine URT.

**Fig 5:**
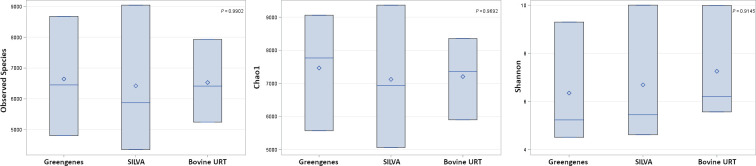
Boxplots of alpha diversity indices among Greengenes, SILVA, and Bovine URT database microbial profiling methods. Alpha diversity indices include A) observed species, B) Chao1, and C) Shannon diversity index.

Fig 4 shows the comparison among taxonomic composition at the phylum level when analyzing short read 16S amplicons against the Greengenes and SILVA database in a de novo pipeline and against the Bovine URT database. The top phyla from the Greengenes analysis at ≥1% included Actinobacteria (27.6%), Firmicutes (23.7%), Bacteroidetes (20.7%), Tenericutes (13.8%), and Proteobacteria (6.1%) (S3 File). The top phyla from SILVA analysis at >1% included Actinobacteria (27.9%), Firmicutes (23.6%), Bacteroidetes (20.8%), Tenericutes (13.7%), and Proteobacteria (6.0%) (S3 File). The top phyla from the Bovine URT database analysis at ≥1% included Actinobacteria (27.9%), Firmicutes (23.8%), Bacteroidetes (21.0%), Tenericutes (14.2%), and Proteobacteria (6.1%) (S3 File). Unassigned taxa in the Bovine URT database analysis was 0.2% contrasted to the Greengenes and SILVA analyses, at 4.2% and 4.0% respectively (S3 File). The unassigned taxa were the only group at the phylum level that differed among the Greengenes (4.2 ± 0.21%), SILVA (4.1 ± 0.24%), or Bovine URT (0.2 ± 0.09%) databases (*P* = 0.0025). When contrasting taxonomic composition, specifically at the genus level, the Bovine URT compositional comparisons against the Greengenes and SILVA taxonomic compositions indicated statistically significant correlations (*r* = 0.226; *P* < 0.001 and *r* = 0.154; *P* < 0.001, respectively). All alpha diversity indices among the three analysis methods were not different (Fig 5). Many sequences and their resultant taxa were successfully assigned by the Bovine URT database but unassigned against the Greengenes database and included those of the order Clostridiales, the family Microbacteriaceae, and genera *Mycoplasma* and *Moraxella*, to name a few (S4 File).

## Discussion

Over the past decade, high-throughput sequencing has dominated microbial ecology and permitted deep investigation into microbial community composition. As a result, numerous databases containing annotated microbial genomes and 16S amplicon reads of numerous microbial ecosystem origins have been constructed, including but not limited to RDP [3], Greengenes [1], SILVA [2], and GenBank [4]. In the typical 16S analysis pipeline, OTU analyses are utilized first, as they are powerful tools in that they do not require a reference database, and function by identifying similar sequences based on sequence identity. Using these databases, taxonomic-dependent methods are then used to assign taxonomic annotations to OTUs based on the OTU analysis. Consequently, the 16S analysis pipeline is limited by the reference database utilized in analyses. Taxonomic annotation of discovered and binned sequences from 16S rDNA read data is a critical step in the canonical 16S amplicon sequencing pipeline. Yet, the use of public databases containing microbial genomic data from numerous microbial ecosystems may not be specific or of high enough quality to properly analyze niche-specific microbial communities. Public 16S databases are popular, but are of variable quality [42, 43] due to their rapid growth and sequence types [44]. The issue is also greater for identification of short reads, commonly used in 16S analyses due to the great depth achieved. With short-read analysis, gaining information beyond the genus level and avoiding mismatches due to the variety of comparative lengths of sequence in the database is often problematic. It is often necessary to increase the taxonomic resolution of microbial community analyses, especially when determining pathology or examining welfare issues, as species-level function and dynamics are essential [45]. The objective of the study was to evaluate the impact of creating a niche-specific, near-full-length, 16S rRNA reference database on the ability to assign taxonomy from shorter partial sequence covering only one to three variable regions. This was completed by analyzing the URT of cattle, a niche with substantial sample to sample variability and relatively high community diversity. Importantly, a niche-specific custom database to be used as a reference also has the disadvantage of being circularly self-referenced and fail to identity taxa that were not present at its setup. These failures could be infrequent but, nevertheless, may be important for the specific habitat. To overcome this consideration for this evaluation, we had available a broad breadth of samples taken at varying locations, environments, breeds, US regions, and time points collected as part of studies on bovine respiratory disease.

The cattle utilized for the development of the Bovine URT database originated from numerous locations and were of varying health status. This variety provided a great resource for broad sampling of URT microbial diversity, as demonstrated in the database phylogenetic tree. Sequences from the phylum Tenericutes and its genus *Mycoplasma* dominated the dataset, which may be due in part to effects of antibiotic treatment of a portion of the animals, or to the stress of transport that places the animal at increased risk for disease (35). Consistent with this supposition, the *Mannheimia* genus was relatively abundant (0.7%). A variety of organisms commonly found in numerous other microbial ecosystems were also identified, expanding the scope of the Bovine URT database for BRD microbial analysis. Organisms in the database that are found in various microbial ecosystems include, but are not limited to, *Prevotella*, commonly found in gastrointestinal tract*s* [46], *Streptobacillus*, commonly found in mammalian disease [47], and *Clostridium*, whose species are found in a wide variety of hosts and have various functions and pathogenicity [46, 48–50]. In all, there were a total of 2,111 observed species, reflecting encounters with diverse environments by the cattle URT as they move from farm to feedlot. Indeed, this diversity reflects the broad sampling of animals across breeds and environments, an important factor in developing a comprehensive, custom database for the cattle URT. The rarefaction curve of the sequences utilized in the Bovine URT database indicates that the level of sequencing depth was adequate to detect the majority of the microbial communities across the dataset. Taking into account the use of near full length 16S reads, deep sequencing depth, variable health statuses, and a large sample population of cattle, the constructed Bovine URT 16S rDNA database represents one of the more comprehensive microbial datasets to date with reference to BRD.

Numerous studies have suggested the use of niche-specific databases for 16S analysis of microbial profiling [12, 29, 51, 52]. In support of this concept, validation of the Bovine URT database was completed using short read amplicons from samples of the USMARC population against the long-read Bovine URT database, as well as against the SILVA and Greengenes databases. Interestingly, the profiling methods referencing the Bovine URT, SILVA, and Greengenes databases produced considerably similar microbial community profiles. Even given the observation of animal-to-animal variation within the long read data, the phylum and genus distribution did not vary greatly between methods, substantiating the utility of the Bovine URT database based on its similarity to the two public databases. However, there was a significant difference in the number of unassigned taxa, which was likely a result of greater classification accuracy. Indeed, these re-assigned taxa using the Bovine URT database likely accounted for some of the small differences observed among the analyses when using the Bovine URT database, such as the phyla Tenericutes, Firmicutes, and Bacteroidetes (S4 File). Nucleotide variation along the 16S rRNA gene is considerable, and short reads can only capture a fraction of this variability. Even when full length reads are analyzed, studies have determined that reads may be taxonomically similar at the genus level. Against the RDP database, one study found as many as 5.5% full-length reads similar at the genus level [53]. Given the variability of sequence length and variability of well-characterized species in public databases, short reads are likely matched to multiple fragments along longer reads of the 16S sequence, resulting in false positives or misclassifications cofounding analyses and exponentially augmenting species estimates, phylogenetic diversity, and taxonomic assignments [54, 55]. Mock community analyses with *de novo* OTU picking strategies have demonstrated inflated OTU counts and diversity [55, 56], and many of the spurious OTUs detected are present at low abundances. This concept was supported by the great discrepancy among analyses in the current study pertaining to the difference in unassigned taxa, as well as the number of genera identified among the three analyses, not to be confused with taxonomic composition and phylogenetic diversity. The Bovine URT analysis identified 550 genera, whereas the number of genera in the SILVA and Greengenes analyses were far greater at 981 and 748 respectively (S3 File). This discprepency is expected to be the result of overestimation due to misclassification to reference sets or low quality assignment [52]. Importantly, during development of the Bovine URT database, the taxonomy was assigned to the long-read sequences using Greengenes. However, this does not imply similar database functionality between the two databases. Once created, the Bovine URT database was unique from the Greengenes database. When short-read samples were mapped to both databases for validation, a difference in results would be expected, as there are different sequences between the databases which can result in the aforementioned differences identified in the current study.

There are common drawbacks and pitfalls inherent to many microbial community surveys. Amplification biases due to unequal matching performance of primers across different species are also considerations of additional sources of error. This can lead to major artifacts in the reported taxa proportions within the community. These primer biases have been reported in our previous work and the work of others [29, 30, 57]. However, in this study, this concern is limited with regard to Bovine URT database validation. We utilized a diverse set of microbial samples for database construction coupled with the same primer pairs among all replicates for validation analyses between two distinct databases. Bovine URT database validation using test sequences among different variable regions may output slightly different results [57], but is beyond the scope and intent of this study. Ultimately, the near-full length read database represents a resource to better interpret the data regardless of primers and hypervariable regions.

Longer reads offer greater potential to identify higher quality OTUs [29], and using these longer reads as a standardized reference is promising to capture accurate microbial composition and diversity. The abundance of unassigned taxa among the methods further supports this theory, and encourages the use of reference databases. The significantly reduced unassigned taxa alludes to a more thorough community composition analysis when assigning short reads to a long read reference database. Moreover, assigning short reads to taxa from a database containing classified long reads that are analyzed and included specifically for the ecosystem studied allowed for a reduction in redundancy and reduced the likelihood that taxa went unassigned, as only 793 of over 170,000 sequences were unable to be identified (0.4%) in the Bovine URT database.

## Conclusions

We were able to demonstrate greater bacterial assignment and reduced unassigned reads using the Bovine URT database developed from near-full-length 16S reads for short-read 16S analyses of URT bacteria in cattle. The analyses utilizing the Bovine URT reference database were also able to identify bacteria previously reported in bovine BRD research demonstrating its effective practice [23–25, 33]. Accurate taxonomic annotation of sequencing reads is imperative for profiling the composition of microbiomes. This is also critical in application, as identifying microbiome dysbioses or pathogens permits researchers with greater opportunities to define the complex interactions in multifactorial disorders, such as BRD. The development of this Bovine URT reference database provides researchers a tool with considerable improvements in performance for the analysis of bacterial communities, and in this study, those of the URT relating to cattle diagnosed with BRD.

## Supporting information

S1 File Species in the reference Bovine URT database. (XLSX)

S2 File Genera in the reference Bovine URT database. (XLSX)

S3 File Phyla and genera compositions from the Greengenes, SILVA, and Bovine URT analyses. (XLSX)

S4 File Taxa that could be assigned by the Bovine URT but not the Greengenes database. (XLSX)
